# Determination of HMGB1 in hepatitis B virus-related acute-on-chronic liver failure patients with acute kidney injury: Early prediction and prognostic implications

**DOI:** 10.3389/fphar.2022.1031790

**Published:** 2023-01-13

**Authors:** Yu Liu, Wei Yuan, Miao Fang, Hongying Guo, Xin Zhang, Xue Mei, Yuyi Zhang, Longshan Ji, Yating Gao, Jiefei Wang, Zhiping Qian, Man Li, Yueqiu Gao

**Affiliations:** ^1^ Laboratory of Cellular Immunity, Institute of Clinical Immunology, Shuguang Hospital Affiliated to Shanghai University of Traditional Chinese Medicine, Shanghai, China; ^2^ Department of Liver Intensive Care Unit, Shanghai Public Health Clinical Center, Fudan University, Shanghai, China; ^3^ Institute of Infectious Diseases of Integrated Traditional Chinese and Western Medicine, Shanghai, China

**Keywords:** hepatitis B virus-related acute-on-chronic liver failure, acute kidney injury, high mobility group protein 1, prediction, prognosis

## Abstract

**Background:** Acute kidney injury (AKI) is a frequent complication in patients with hepatitis B virus-related acute-on-chronic liver failure (HBV-ACLF) and is associated with high rates of mortality. We aimed to estimate serum high mobility group protein 1 (HMGB1) levels in hepatitis B virus-related acute-on-chronic liver failure patients and analyze their clinical value in the development and outcomes of Acute kidney injury.

**Methods:** A total of 251 consecutive patients with hepatitis B virus-related acute-on-chronic liver failure were enrolled in this retrospective study. Using the International Club of Ascites staging criteria of Acute kidney injury, 153 patients developed Acute kidney injury. The clinical data of patients were collected and serum levels of high mobility group protein 1 were measured by ELISA. All patients were followed up until death or for a minimum of 3 months. Early prediction and prognostic implications of high mobility group protein 1 in Hepatitis B Virus-Related Acute-on-Chronic Liver Failure Patients with Acute Kidney Injury were investigated in different cohorts, including a propensity score-matched ACLF cohort.

**Results:** Among all individuals with hepatitis B virus-related acute-on-chronic liver failure, the incidence of Acute kidney injury was 61.0% (153/251). The patients who developed stage 2/3 Acute kidney injury showed the highest high mobility group protein 1 levels, followed by those who developed stage 1 Acute kidney injury, and those without Acute kidney injury showed the lowest high mobility group protein 1 levels. Moreover, high mobility group protein 1 levels were significantly higher in non-survivors than in survivors among hepatitis B virus-related acute-on-chronic liver failure patients with Acute kidney injury. Furthermore, analysis of the area under the receiver operating characteristic curve (AUROC) indicated that serum high mobility group protein 1 levels (pre-matching: AUC = 0.740; post-matching: AUC = 0.661) may be a potential predictive factor for Acute kidney injury development and that high mobility group protein 1 (AUC = 0.727) might be a reliable biomarker for prognosis in patients with Acute kidney injury.

**Conclusion:** In patients with hepatitis B virus-related acute-on-chronic liver failure, Acute kidney injury is universal. Acute kidney injury and its stages negatively influence the 90-day transplant-free mortality rate. Serum high mobility group protein 1 levels can serve as a positive predictor of Acute kidney injury development, and high mobility group protein 1 might also be a prognostic biomarker for Acute kidney injury among hepatitis B virus-related acute-on-chronic liver failure patients.

## Highlights

1) Elevated HMGB1 levels correlated with AKI stage in patients with HBV-ACLF.
2) HMGB1 can be used as an early AKI predictor in individuals with HBV-ACLF.
3) HMGB1 can serve as a prognostic biomarker for AKI in individuals with HBV-ACLF.

## Introduction

Acute kidney injury (AKI) is a major clinical concern of acute-on-chronic liver failure (ACLF). In China, patients with hepatitis B virus (HBV)-related acute-on-chronic liver failure (HBV-ACLF) are characterized by high-level systemic inflammation, organ failure and high 90-day mortality (50%–70%) (Li et al., 2016; Wu et al., 2018). The occurrence of AKI in ACLF usually aggravates the disease and frequently leads to a negative prognostic impact. A recent meta-analysis concluded that approximately 40% of ACLF patients had AKI complications and that AKI is substantially associated with increased short-term mortality (Jiang et al., 2020). The identification of AKI is therefore of great importance for prevention and early treatment. Early prediction of AKI development provides a critical therapeutic time window and will provide the most appropriate and timely interventions for patients, which may contribute to better outcomes for patients with AKI. However, the current clinical detection based on creatinine and urine output (UO) is not effective in the early detection of AKI. AKI diagnosis is currently based on an increase in serum creatinine (sCr) and weight-adjusted hourly UO. Unfortunately, Cr is unreliable for predicting acute changes in kidney function. Over the past decades, researchers have evaluated more than 26 new biomarkers for AKI, including cystatin C (CysC) (Jha et al., 2021), neutrophil gelatinase-associated lipocalin (Nickolas et al., 2012), angiotensinogen (Chen C et al., 2016), and liver-type fatty acid-binding protein (Ferguson et al., 2010). However, the high cost, instability and limitations of these markers hinder their wide application in clinical and biochemical settings, and most of these markers need continuous systemic evaluation, which is not cost-effective. To date, only CysC has small-scale clinical applications.

High mobility group box 1 (HMGB1), a proinflammatory cytokine, was discovered several decades ago (Goodwin et al., 1973) and is one of the most abundant and highly conserved proteins in eukaryotic cells. HMGB1 regulates cellular proliferation, differentiation, tissue regeneration and inflammation (Gu et al., 2016), which contributes to kidney disease pathogenesis. The pathogenic mechanism for AKI in patients with ACLF may be related to bacterial endotoxin, inflammatory cytokines, and reduction of effective renal blood volume caused by substantial ascites. A cross-sectional study showed that serum HMGB1 levels increased in AKI patients and that elevated HMGB1 levels were positively associated with leukocyte count and negatively associated with proteinuria (Zakiyanov et al., 2013). Infection accompanied by the release of HMGB1 contributes to circulatory dysfunction and is one of the major contributing factors of AKI. Experimental studies have shown that HMGB1 release from podocytes is induced by lipopolysaccharide (LPS), which subsequently exacerbates AKI (Gao et al., 2021). The release of HMGB1 worsened circulatory dysfunction. Recently, a study showed that serum HMGB1 levels had a high correlation with infection and a moderate correlation with AKI and death (Vilela et al., 2018). Another study including 25 cirrhotic patients with AKI demonstrated that the presence of high HMGB1 levels was associated with decreased survival (de Oliveira Gomes et al., 2020). Therefore, serum HMGB1 levels may be associated with the development or progression of AKI. Moreover, the aberrant expression of HMGB1 may also be related to worse prognosis in AKI patients.

AKI is a common and serious complication of HBV-ACLF. However, comparatively little is known about the impact of HMGB1 on ACLF-associated AKI. In this study, we measured the serum levels of HMGB1 in all enrolled patients, analyzed the clinical relevance of HMGB1 levels to ACLF-associated AKI and further explored the early predictive and prognostic values of HMGB1 in ACLF-associated AKI.

## Methods

### Study patients and design

**FIGURE 1:**
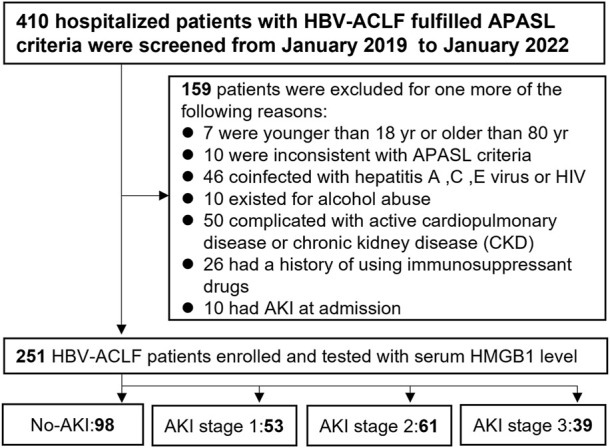
Flow chart of all enrolled HBV-ACLF patients with AKI after applying the inclusion and exclusion criteria.

From January 2019 through January 2022 at Shanghai Public Health Clinical Center, 410 hospitalized patients with HBV-ACLF were reviewed in this retrospective study. The flow chart is shown in Figure 1. In light of the unanimous suggestions of the Asian Pacific Association for the Study of the Liver (APASL) (Sarin et al., 2019), patients matching the following criteria were recruited for this research: 1) a history of HBV-related cirrhosis or chronic liver disease that was either previously recognized or gone undetected; 2) 18–80 years old; 3) coagulation abnormalities [prothrombin activity ≤40% or international normalized ratio (INR) ≥ 1.5] with serum total bilirubin (TBIL) ≥ 5 mg/dl (85 µmol/L); and 4) ascites and hepatic encephalopathy (HE) clinical manifestations within 4 weeks. Patients were excluded if they had one of exclusion criteria: 1) inconsistent with APASL criteria; 2) co-infection with the human immunodeficiency virus or the hepatitis A/C/E virus; 3) patients who had other chronic liver diseases; 4) patients who had severe extra-hepatic diseases; 5) patients who had previously used hepatotoxic, immunosuppressive, or anticancer medications; 6) patients who had severe disease complications which may affect survival time; 7) patients who had AKI at admission; 8) pregnancy. Finally, this research included 251 individuals with HBV-ACLF. The baseline data and demographic information for participants at admission were collected, including demographic data, complication rate, and laboratory measures. Peripheral blood was collected at baseline to measure HMGB1 levels. In addition, detailed methods of score calculation are specified in the Supplemental Methods, including the model for end-stage liver disease (MELD), Child-Turcotte-Pugh (Child-Pugh), CLIF Consortium Organ Failure (CLIF-OF) and CLIF-Consortium ACLF (CLIF-C ACLF) scores. Based on the established guidelines (Sarin et al., 2009; European Association for the Study of the Liver, 2010; Sarin et al., 2014; Vilstrup et al., 2014), patients with HBV-ACLF were managed.

### Definition of AKI or renal response to treatment

AKI diagnoses and classification were performed in accordance with the International Club of Ascites (ICA) (Angeli et al., 2015). We evaluated renal function at baseline based on the most recent sCr level within 3 months prior to admission or the sCr value measured first after hospitalization. AKI was defined as follows: AKI stage 1 was defined as sCr increased by ≥ 0.3 mg/dl (26.5 µmol/L) in 48 h or to 1.5–2.0 times baseline during the first 7 days; AKI stage 2 was defined as sCr increased 2.0–3.0 times from baseline; and AKI stage 3 was defined as sCr increased 3.0 times from baseline or an increase by ≥ 4.0 mg/dl (353.6 μmol/L) or initiation of renal replacement treatment (RRT).

To compare the renal outcome among different stages of AKI, we made the following definition. We defined Stage 1 (0.3) as an increase of ≥0.3 mg/dl in the sCr level in 48 h only; Stage 1 (50%) as a 50% increase above baseline sCr; Stage 3 as not requiring RRT; and Stage 3 (RRT) as a requirement of RRT.

The peak sCr was defined by the highest sCr value during hospital admission. We defined stage 2–3 AKI as severe AKI. The maximum AKI stage (AKI_max_) was abstracted from medical records during hospitalization.

According to the ICA, renal responses to treatment were classified as complete response (CR), partial response (PR), or absence of response (AR). CR represents an sCr return to within 0.3 mg/dl at hospital discharge. PR represents a decrease in AKI stage compared with their maximal AKI stage or a sCr reduction to ≥0.3 mg/dl. AR represents without recovery compared with their maximal AKI stage.

### Study end points

All patients were followed up until death or at least 3 months. Our primary outcome was 90-day survival. Liver transplantation (LT) or death was considered the adverse endpoint. Our secondary outcome measure was AKI outcome (CR, PR, AR) defined according to ICA criteria.

### Measurement of serum HMGB1

Quantification of HMGB1 in plasma was performed using ELISA kits (Cat. ST51011, Shino-test Corporation, Kanagawa, Japan). The assay was conducted in accordance with the manufacturers’ instructions. The specimens were stored in a -80°C freezer until further handling.

### Statistical analysis

In this study, normally distributed continuous variables are expressed as the mean ± SD, and categorical variables are expressed as frequencies and percentages. Quantitative variables were compared using Student’s t test, and categorical variables were compared using the chi-square test or Fisher’s exact test as appropriate.

Continuous variables that were not normally distributed are presented as the median (interquartile range [IQR]) and were compared by using Mann-Whitney U tests or Kruskal–Wallis tests. Continuous variables of three or more groups were analyzed using the Kruskal–Wallis test. Propensity score–matching analysis was used to reduce the effect of selection bias and potential confounding between the no-AKI and AKI groups. For propensity score matching, a nearest-neighbor 1:1 matching scheme with a caliper size of 0.02 was used. Survival analyses were performed using the Kaplan-Meier method and the log-rank test. Multivariate analysis was carried out using Cox regression models and logistic regression. The HMGB1 levels were divided into four groups according to the HMGB1 quartile levels (negative: <25%; low: 25–49.9%; moderate: 50–74.9%; high: ≥75%), and the *p*-value for trend was obtained by regression analysis. The performance of HMGB1 was assessed by the area under the receiver operating characteristic curve (AUC). To identify a differential effect of HMGB1 on outcomes per subgroup, interaction analysis using the Cox model was conducted, where an interaction term was composed of HMGB1 and a subgroup variable and presented with forest plots.

All statistical analyses were performed with SPSS 25.0 (IBM, Seattle, WA, USA) software or in R (version 4.0.4, http://www.r-project.org). Two-sided *p* < 0.05 was considered statistically significant.

## Results

### Demographic and clinical characteristics of the participants

A total of 251 patients with HBV-ACLF were enrolled. A total of 153 (61.0%) of 251 patients with HBV-ACLF developed AKI after hospital admission. Among those with AKI, 53 patients (34.6%) had stage 1 AKI, 61 patients (39.9%) had stage 2 AKI, and 39 patients (25.5%) had stage 3 AKI. The clinical baseline characteristics of the no-AKI and AKI stage 1–3 groups in the present study are shown in Table 1. The patients with AKI were older than patients without AKI (*p* = 0.001). The patients with AKI had higher TBIL (*p* < 0.001), INR (*p* = 0.035), HBVDNA (*p* = 0.046), sCr (*p* < 0.001), peak sCr (*p* < 0.001), CysC (*p* < 0.001), WBC (*p* = 0.012), and serum lactate (*p* = 0.023) levels, and lower Na (*p* = 0.008) levels than the patients without AKI. Patients with AKI more frequently presented with HE (*p* = 0.001), infection (*p* = 0.001) and ascites (*p* = 0.024) at admission than patients without AKI. Compared with individuals without AKI, individuals with AKI had higher MELD (*p* < 0.001), Child–Pugh (*p* < 0.001) and CLIF-C ACLF (*p* < 0.001) scores.

**TABLE 1:** Baseline demographic and clinical parameters of HBV-ACLF patients with and without AKI according to ICA criteria.

Characteristics	AKI (n = 153)				
	No-AKI (n = 98)	stage 1 (n = 53)	stage 2 (n = 61)	stage 3 (n = 39)	*p-*value
Demographics					
Age (years)	44 [37–54.25]	47 [39–55.5]	53 [42–61]	52 [43–66]	0.001
Male (n, %)	85 (86.7)	47 (88.7)	54 (88.5)	34 (87.2)	0.981
Cirrhosis (n, %)	45 (45.9)	31 (58.5)	30 (49.2)	24 (61.5)	0.266
Acute decompensation					
HE					0.001
No HE	82 (83.7)	42 (79.2)	39 (63.9)	18 (46.2)	
grade 1–2	10 (10.2)	7 (13.2)	12 (19.7)	11 (28.2)	
grade 3–4	6 (6.1)	4 (7.5)	10 (16.4)	10 (25.6)	
Infection	60 (61.2)	32 (60.4)	50 (82)	35 (89.7)	0.001
Ascites	58 (59.2)	37 (69.8)	47 (77)	32 (82.1)	0.024
GI bleeding	5 (5.1)	2 (3.8)	7 (11.5)	3 (7.7)	0.337
Laboratory tests					
ALT (U/L)	190.5 [57.5–755.75]	190 [83.5–766]	213 [103–569]	118 [67–491]	0.262
AST (U/L)	135.5 [70.25–507.5]	160 [78–426]	217 [113.5–450]	122 [73–217]	0.051
CK (U/L)	63 [42–107.25]	73 [51.5–123.5]	74 [49.5–124.5]	100 [49–213]	0.058
TBIL (mg/dl)	16.18 [10.64–23.35]	18.23 [12.06–26.56]	25.91 [13.87–32.53]	24.21 [18.13–31.07]	<0.001
ALB (g/L)	31.77 [29.05–34.2]	31 [26.96–33.29]	29.93 [27.17–34.44]	30.35 [27.12–33.87]	0.280
INR	2.02 [1.75–2.52]	2.33 [1.87–2.64]	2.36 [1.77–3.18]	2.34 [1.84–2.98]	0.035
log10HBVDNA (IU/ML)	2.49 [1.9–4.09]	3.21 [1.9–5.25]	4.04 [2.93–5.31]	2.66 [2.08–5.07]	0.046
HBeAg positive (n, %)	41 (41.8)	14 (26.4)	28 (45.9)	13 (33.3)	0.133
sCr (mg/dl)	0.74 [0.65–0.94]	0.95 [0.7–1.2]	0.98 [0.87–1.22]	1.02 [0.71–1.21]	<0.001
Peak sCr (mg/dl)	0.86 [0.72–0.96]	1.36 [1.07–1.79]	2.24 [1.92–2.71]	4.19 [3.17–5.5]	<0.001
CysC (mg/dl)	1.13 [0.93–1.34]	1.26 [1.1–1.5]	1.49 [1.22–2.39]	1.57 [1.35–2.52]	<0.001
WBC (10^9/L)	6.41 [4.66–8.17]	5.21 [4.14–8.31]	6.53 [5.13–10.22]	7.91 [5.81–11.32]	0.012
PLT (10^9/L)	89 [50–132.5]	78 [57–119]	84 [58.5–118]	89 [60–116]	0.800
Serum lactate (mmol/L)	2.66 [2.09–3.26]	2.54 [2.04–3.14]	2.88 [2.33–3.68]	2.93 [2.52–3.85]	0.023
Na (mmol/L)	136 [134–139]	135 [133–138]	135 [131.5–137]	133 [130–137]	0.008
HMGB1 (ng/ml)	2.67 [1.74–3.63]	3.46 [2.54–4.9]	4.62 [2.74–6.6]	6.49 [3.32–9.84]	<0.001
Scores					
MELD	22.44 [20.03–25.56]	25.6 [23.15–27.79]	28.14 [23.62–30.99]	26.92 [24.08–30.2]	<0.001
Child-Pugh	11 [10–12]	11 [11–12]	11 [11–13]	12 [11–13]	<0.001
CLIF-C ACLF	40.57 [36.18–45.59]	43.39 [38.92–49.43]	47.72 [39.91–54.5]	50.12 [42.27–57.77]	<0.001
Outcomes(death/survival/LT)					
90-day	10/83/5	11/34/8	28/23/10	31/7/1	<0.001

HE, hepatic encephalopathy; GI, gastrointestinal; ALT, alanine aminotransferase; AST, aspartate aminotransferase; CK, creatine kinase; TBIL, total bilirubin; ALB, albumin; INR, international normalized ratio; sCr, serum creatinine; CysC, cystatin C; WBC, white blood cell; PLT, platelet; Na, serum sodium; HMGB1, high-mobility group box 1; MELD, model for end-stage liver disease; Child-Pugh, Child-Turcotte-Pugh; CLIF-C ACLF, CLIF-Consortium ACLF; LT, liver transplantation.

The serum levels of HMGB1 in patients with stage 3 AKI [6.49 [3.32–9.84] mg/dl] and stage 2 AKI [4.62 [2.74–6.6] mg/dl] were meaningfully higher than those with stage 1 AKI [3.46 [2.54–4.9] mg/dl], and serum HMGB1 levels in patients with stage 1 AKI were relatively higher than those without AKI [2.67 [1.74–3.63] mg/dl] (Table 1 and Supplementary Figure S1A). However, no significant difference was observed between the AKI stage 3 and AKI stage 2 groups (*p* = 0.193, Supplementary Figure S1A). To elucidate the clinical significance of HMGB1 for ACLF, we compared serum HMGB1 levels among HBV-ACLF patients between different groups of organ failure according to CLIF-C OF scores. A positive correlation was found between serum HMGB1 levels and the degrees of brain failure or coagulation failure but not liver failure (Supplementary Figure S1B, S1C, S1D).

To evaluate whether HMGB1 levels constitute a risk factor independent of early AKI prediction in HBV-ACLF patients, as confounding covariates, we included age, ascites, CysC, sCr, and CLIF-C ACLF scores and performed 1:1 propensity score matching (n = 138, 69 patients with AKI were matched to 69 patients without AKI). Baseline characteristics of pre- and post-matching cohorts are displayed in Table 2. Hospitalized HBV-ACLF patients who experienced AKI had significantly higher baseline HMGB1 levels than well-matched patients hospitalized without AKI (*p* = 0.001).

**TABLE 2:** Characteristics of 251 pre-matching or 138 post-matching ACLF patients (1:1 propensity score matching (No-AKI: AKI), covariables age, ascites, CysC, sCr, and CLIF-C ACLF score).

Characteristics	Pre-matching			Post-matching		
	No-AKI (n = 98)	AKI (n = 153)	*p*-value	No-AKI (n = 69)	AKI (n = 69)	*p*-value
Demographics						
Age (years)	45.69 ± 12.30	51.00 ± 12.50	0.001	46.52 ± 12.14	46.62 ± 10.75	0.959
Male (n, %)	85 (86.7)	135 (88.2)	0.724	62 (89.9)	62 (89.9)	1.000
Cirrhosis (n, %)	45 (45.9)	85 (55.6)	0.136	34 (49.3)	37 (53.6)	0.609
Acute decompensation						
HE	16 (16.3)	54 (35.3)	0.001	13 (18.8)	22 (31.9)	0.078
Infection	60 (61.2)	117 (76.5)	0.010	44 (63.8)	51 (73.9)	0.198
Ascites	58 (59.2)	116 (75.8)	0.005	46 (66.7)	48 (69.6)	0.715
GI bleeding	5 (5.1)	12 (7.8)	0.399	5 (7.2)	5 (7.2)	1.000
Laboratory tests						
ALT (U/L)	190.5 [57.5–755.75]	190 [87–553]	0.662	149 [59.5–495]	147 [67–453.5]	0.790
AST (U/L)	135.5 [70.25–507.5]	160 [100.5–381.5]	0.250	136 [78.5–334.5]	143 [81–303.5]	0.782
CK (U/L)	63 [42–107.25]	78 [50–129]	0.045	62 [41.5–105.5]	76 [51.5–126.5]	0.208
TBIL (mg/dl)	16.18 [10.64–23.35]	22.23 [14.2–30.69]	<0.001	17.84 [12.29–24.06]	18.63 [10.52–28.58]	0.378
ALB (g/L)	31.77 [29.05–34.2]	30.35 [27.14–33.86]	0.052	31.46 [29.1–34.25]	30.79 [26.74–34.12]	0.271
INR	2.02 [1.75–2.52]	2.34 [1.85–2.97]	0.004	2.12 [1.73–2.66]	2.1 [1.74–2.67]	0.586
log10HBVDNA (IU/ML)	2.49 [1.9–4.09]	3.37 [1.9–5.14]	0.052	2.4 [1.9–3.7]	2.81 [1.9–4.74]	0.570
HBeAg positive (n, %)	41 (41.8)	55 (35.9)	0.349	30 (43.5)	21 (30.4)	0.112
sCr (mg/dl)	0.74 [0.65–0.94]	0.96 [0.72–1.21]	<0.001	0.86 [0.67–0.95]	0.75 [0.66–0.97]	0.775
CysC (mg/dl)	1.13 [0.93–1.34]	1.42 [1.15–2.02]	<0.001	1.18 [0.97–1.39]	1.27 [1.08–1.54]	0.064
WBC (10^9/L)	6.41 [4.66–8.17]	6.77 [4.79–9.77]	0.190	6.57 [4.76–9.04]	5.73 [4.06–8.21]	0.136
PLT (10^9/L)	89 [50–132.5]	82 [59–118]	0.510	88 [49–131]	81 [57.5–115.5]	0.614
Serum lactate (mmol/L)	2.66 [2.09–3.26]	2.78 [2.23–3.42]	0.232	2.75 [2.15–3.31]	2.62 [2.17–3.15]	0.626
Na (mmol/L)	136 [134–139]	135 [132–137]	0.005	136 [134–139]	135 [132–137]	0.056
HMGB1 (ng/ml)	2.67 [1.74–3.63]	4.48 [2.86–6.29]	<0.001	2.63 [1.87–4.18]	4.04 [2.65–5.08]	0.001
Scores						
MELD	22.44 [20.03–25.56]	26.47 [23.47–30.15]	<0.001	23.34 [21.36–26.38]	23.88 [21.78–27.33]	0.280
Child-Pugh	11 [10–12]	12 [11–13]	<0.001	11 [10–12]	11 [11–12.5]	0.063
CLIF-C ACLF	40.57 [36.18–45.59]	45.6 [40.28–53.06]	<0.001	42.16 [37.06–46.81]	41.67 [36.53–47.8]	0.751

HE, hepatic encephalopathy; GI, gastrointestinal; ALT, alanine aminotransferase; AST, aspartate aminotransferase; CK, creatine kinase; TBIL, total bilirubin; ALB, albumin; INR, international normalized ratio; sCr, serum creatinine; CysC, cystatin C; WBC, white blood cell; PLT, platelet; Na, serum sodium; HMGB1, high-mobility group box 1; MELD, model for end-stage liver disease; Child-Pugh, Child-Turcotte-Pugh; CLIF-C ACLF, CLIF-Consortium ACLF; LT, liver transplantation.

### Clinical characteristics of non-survivors and survivors in AKI individuals with HBV-ACLF

According to the 90-day prognosis, the AKI group was divided into a survival group (n = 64) and a non-survival group (n = 89). We found that non-survivors were older than survivors (*p* = 0.044). Meanwhile, non-survivors had a higher frequency of HE (*p*<0.001), infection (*p* = 0.001) and ascites (*p* = 0.001). More importantly, the levels of TBIL (*p* < 0.001), INR (*p* = 0.010), peak sCr (*p* < 0.001), serum lactate (*p* = 0.015) and HMGB1 (*p* < 0.001) in non-survivors were also significantly higher than those in survivors. Likewise, the MELD (*p* = 0.001), Child-Pugh (*p* < 0.001) and CLIF-C ACLF (*p* < 0.001) scores were higher at baseline for the non-survival group than for the survival group. The above characteristics in non-survivors and survivors are listed in Table 3.

**TABLE 3:** Baseline characteristics of HBV-ACLF patients with AKI based on different prognostic groups at admission.

Characteristics	Survivors (n = 64)	Non-survivors (n = 89)	*p*-value
Demographics			
Age (years)	47.5 (39–56)	52 (42–64)	0.044
Male (n, %)	57 (89.1)	78 (87.6)	0.788
cirrhosis (n, %)	31 (48.4)	54 (60.7)	0.133
Acute decompensation			
HE			<0.001
No HE	55 (85.9)	44 (49.4)	
grade 1–2	7 (10.9)	23 (25.8)	
grade 3–4	2 (3.1)	22 (24.7)	
Infection	40 (62.5)	77 (86.5)	0.001
Ascites	37 (57.8)	79 (88.8)	0.001
GI bleeding	2 (3.1)	10 (11.2)	0.066
Laboratory tests			
ALT (U/L)	156.5 [80.25–456.75]	228 [87.5–695.5]	0.200
AST (U/L)	143 [80.25–309]	174 [110.5–435.5]	0.057
CK (U/L)	75.5 [50.25–131]	84 [49.5–129]	0.871
TBIL (mg/dl)	16.31 [10.45–26.61]	26.59 [17.37–33.46]	<0.001
ALB (g/L)	30.14 [26.09–33]	30.47 [27.51–34.09]	0.244
INR	2.12 [1.7–2.65]	2.39 [2.02–3.16]	0.010
log10HBVDNA (IU/ML)	3.43 [2.19–5.49]	3.34 [1.9–5]	0.425
HBeAg positive (n, %)	24 (37.5)	31 (34.8)	0.734
sCr (mg/dl)	0.97 [0.79–1.22]	0.96 [0.72–1.21]	0.703
Peak sCr (mg/dl)	1.84 [1.3–2.44]	2.46 [1.71–3.65]	<0.001
CysC (mg/dl)	1.32 [1.10–1.98]	1.44 [1.22–2.04]	0.127
WBC (10^9/L)	6.38 [4.68–9.32]	6.86 [5.02–10.54]	0.388
PLT (10^9/L)	88 [60–115.25]	79 [58–119]	0.727
Serum lactate (mmol/L)	2.6 [2.1–3.15]	2.93 [2.3–3.85]	0.015
Na (mmol/L)	135 [132.25–138]	134 [132–137]	0.166
HMGB1 (ng/ml)	3.09 [2.08–4.96]	5.23 [3.32–8.19]	<0.001
Scores			
MELD	25.21 [21.65–28.56]	27.63 [25.28–30.99]	0.001
Child-Pugh	11 [10.25–12]	12 [11–13]	<0.001
CLIF-C ACLF	42.93 [38.59–47.64]	49.70 [42.39–57.38]	<0.001

HE, hepatic encephalopathy; GI, gastrointestinal; ALT, alanine aminotransferase; AST, aspartate aminotransferase; CK, creatine kinase; TBIL, total bilirubin; ALB, albumin; INR, international normalized ratio; sCr, serum creatinine; CysC, cystatin C; WBC, white blood cell; PLT, platelet; Na, serum sodium; HMGB1, high-mobility group box 1; MELD, model for end-stage liver disease; Child-Pugh, Child-Turcotte-Pugh; CLIF-C ACLF, CLIF-Consortium ACLF; LT, liver transplantation.

### Longitudinal follow-up evaluation and kidney outcomes

The percentages of outcome events (death, survival, LT) between the no-AKI and AKI stage 1/2/3 groups during the 90-day follow-up period in all HBV-ACLF patients are shown in Supplementary Figure S2. AKI patients had a higher LT-free mortality or incidence of adverse outcomes than those without AKI at 90 days (45.8% vs. 10.2%, *p* < 0.001; 58.2% vs. 15.3%, *p* < 0.001).

**FIGURE 2:**
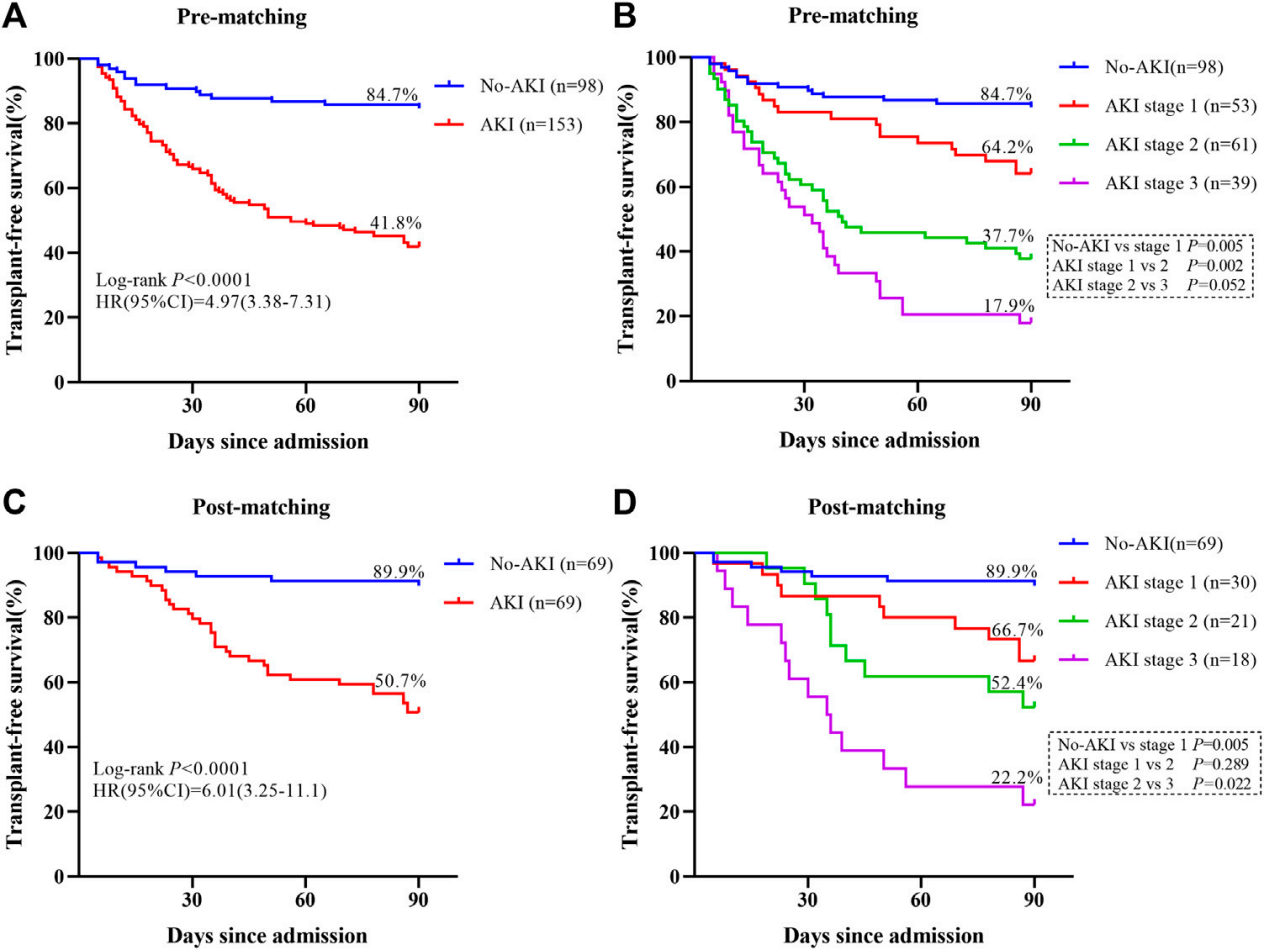
Survival curves for patients with adverse outcomes are shown. **(A)** The Kaplan–Meier curves are classified according to the presence or absence of AKI during the 90-day follow-up period in the pre-matching cohort. **(B)** The Kaplan–Meier curves are classified according to the presence or absence of AKI and its stage during the 90-day follow-up period in the pre-matching cohort. **(C)** The Kaplan–Meier curves are classified according to the presence or absence of AKI during the 90-day follow-up period in the post-matching cohort. **(D)** The Kaplan–Meier curves are classified according to the presence or absence of AKI and its stage during the 90-day follow-up period in the post-matching cohort.

Subsequently, the relationship between different AKI stages and the survival rate was analyzed by using Kaplan-Meier survival curves plotted using 90-day prognosis. Transplant-free survival rates were significantly decreased in patients who developed AKI compared with those who did not develop AKI at 90 days (pre-matching: 41.8% vs. 84.7%, *p* < 0.0001, Figure 2A **;** post-matching: 50.7% vs. 89.9%, *p* < 0.0001, Figure 2C). The transplant-free survival rate of patients without AKI was significantly higher than that of patients with Stage 1AKI (pre-matching: 84.7% vs. 64.2%, *p* < 0.01, Figure 2B **;** post-matching: 89.9% vs. 66.7%, *p* < 0.01, Figure 2D). The transplant-free survival rate was substantially higher among patients in the Stage 1 AKI group than among patients in the Stage 2 AKI group (64.2% vs. 37.7%, *p* < 0.01, Figure 2B) and there was no significant difference in the transplant-free survival rates between patients with Stage 2 AKI and those with Stage 3 AKI in the pre-matching cohort (*p* > 0.05, Figure 2B). The transplant-free survival rate was substantially higher among patients in the Stage 2 AKI group than among patients in the Stage 3 AKI group (52.4% vs. 22.2%, *p* < 0.05, Figure 2D) and there was no significant difference in the transplant-free survival rates between patients with Stage 1 AKI and those with Stage 2 AKI in the post-matching cohort (*p* > 0.05, Figure 2D).

As expected, there was a correlation between kidney outcomes and AKI stages in the pre-matching cohort. Among patients who developed AKI, 17 (11.1%) patients developed Stage 1 (0.3), 36 (23.5%) patients developed Stage 1 (50%), 61 (39.9%) patients developed Stage 2, 30 patients (19.6%) developed Stage 3, and nine patients (5.9%) developed Stage 3 (RRT) (Supplementary Table S1). The CR was only 37.3% in the entire AKI population, 20.3% of patients still had stage 1 AKI, 23.5% of patients still had stage 2 AKI, and 19% of patients had stage 3 AKI. These results are shown in Supplementary Table S1. We found that the CR rate decreased with increasing AKI stage in the entire AKI cohort [i.e., from 76.5% in Stage 1 (0.3) to 22.2% in Stage 3 (RRT) (*p* < 0.001)]. The renal outcomes of surviving patients are listed in Supplementary Table S2. The CR was 64.1%, PR was 12.5% and AR was 23.4% among survivors. The incidence of CR was markedly higher in the surviving patients than in the total AKI population (64.1% vs. 37.3%, *p* < 0.001).

### Performance of serum HMGB1 levels for predicting AKI development

**FIGURE 3:**
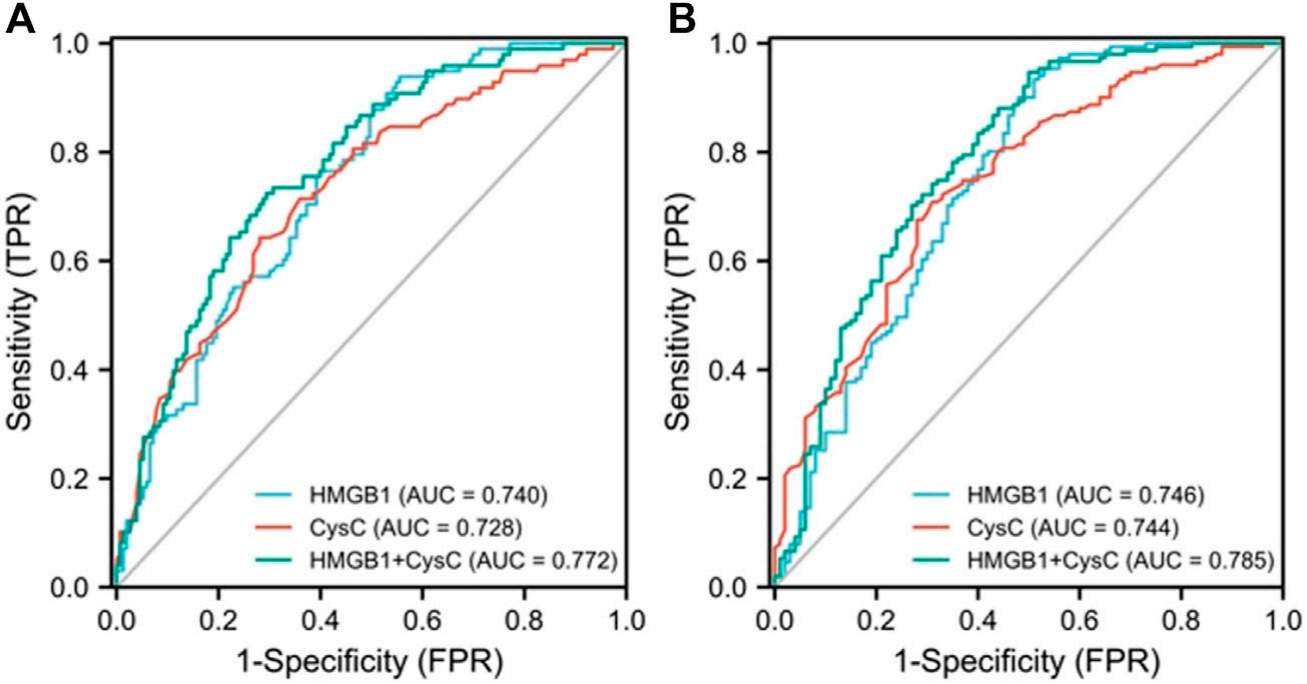
AUROC analysis was performed to compare the efficacy of serum HMGB1 and CysC levels in predicting the development of AKI **(A)** or severe AKI **(B)** in pre-matching HBV-ACLF patients during the 90-day follow-up period (AUROC, area under the receiver operating characteristic curve; HBV-ACLF, hepatitis B virus-related acute-on-chronic liver failure; AKI, acute kidney injury; CysC, cystatin C; HMGB1, high-mobility group box 1).

**FIGURE 4:**
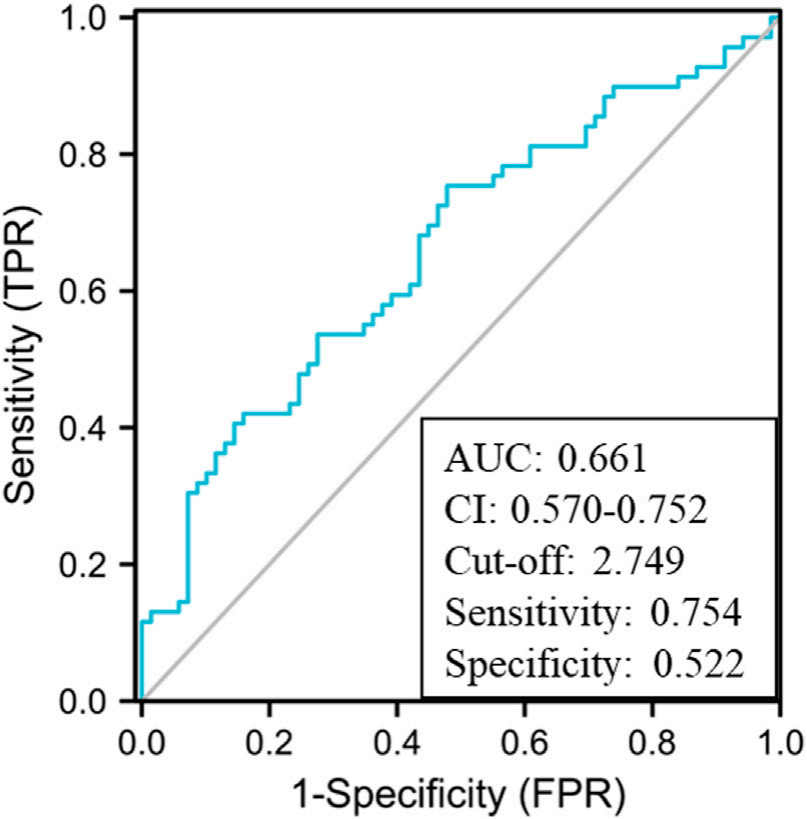
AUROC analysis of serum HMGB1 levels in predicting the development of AKI in the post-matching HBV-ACLF cohort during the 90-day follow-up period (AUROC, area under the receiver operating characteristic curve; HBV-ACLF, hepatitis B virus-related acute-on-chronic liver failure; AKI, acute kidney injury; HMGB1, high-mobility group box 1).

First, an AUROC analysis was used to evaluate the performance of HMGB1 and CysC in predicting the development of AKI in the pre-matching cohort as shown in Figure 3A. Serum HMGB1 showed strong predictive ability, with a high AUROC value [0.740 (95% CI: 0.680–0.800)], which was comparable to the AUROC value obtained with CysC [0.728 (95% CI: 0.664–0.791)]. The joint indicator (HMGB1+ CysC) showed stronger predictive ability [0.772 (95% CI: 0.715–0.830)] than serum HMGB1 (*p* = 0.012). HMGB1 as a potential biomarker predicted AKI development with a sensitivity of 93.9% and specificity of 44.4%, in which the cut-off value was set at 4.850 ng/ml (Supplementary Table S3). Likewise, serum HMGB1 showed strong predictive ability, with a high AUROC value [0.661 (95% CI: 0.570–0.752)] in the post-matching cohort, as shown in Figure 4.

Next, we further explored the potential value of HMGB1 in predicting the development of severe AKI in all enrolled HBV-ACLF patients as shown in Figure 3B. Serum HMGB1 showed strong predictive ability, with a high AUROC value [0.746 (95% CI: 0.680–0.812)], which was comparable to the AUROC value obtained with CysC [0.744 (95% CI: 0.682–0.806)]. The joint indicator (HMGB1+ CysC) showed stronger predictive ability [0.785 (95% CI: 0.724–0.845)] than serum HMGB1 (*p* = 0.007). Furthermore, we further explored the potential predictive value of HMGB1 in the non-cirrhotic (n = 121) and cirrhotic subgroups (n = 130) of all HBV-ACLF patients as shown in Supplementary Figure S3. In the non-cirrhotic or cirrhotic subgroups, HMGB1 and CysC had certain early predictive value for AKI development in HBV-ACLF patients, and there was no statistically significant difference between the two single indicators. In the non-cirrhotic subgroup, HMGB1 and CysC had greater predictive value as joint indicators than serum HMGB1 (*p* = 0.004). Concurrently, in the cirrhotic subgroup, HMGB1 and CysC had greater predictive value as joint indicators than serum CysC (*p* = 0.039). Partial ROC-related information and data for each predictor variable are shown in Supplementary Table S3 and Supplementary Table S4.

Finally, given the essential role of HMGB1 in the development of AKI, we sought to analyze the possible relationship through three-step adjustment by building unadjusted or adjusted models. Model I unadjusted; Model II adjusted for age; Model III adjusted for the covariates in Model II plus HE, infection, and ascites; Model IV adjusted for the covariates in Model III plus Na, TBIL, and INR. These selected covariates were identified through the univariate linear regression analysis in Table 1. After adjusting for the effects of several potential confounders, the role of HMGB1 was not meaningfully affected, with each increase in serum HMGB1 levels, there was an increased risk of AKI development (*p*-value for trend = 0.005) as shown in Table 4.

**TABLE 4:** Adjusted effects of HMGB1 level as a predictor of AKI among individuals with HBV-ACLF during the 90-day follow-up period (n = 251).

HMGB1	Incidence of AKI (n, %)	Or, 95% CI	Or, 95% CI	Or, 95% CI	Or, 95% CI
		*p*-value	*p*-value	*p*-value	*p*-value
		Model I	Model II	Model Ⅲ	Model Ⅳ
HMGB1 (continuous)	153 (61.0)	1.68 (1.4.2.01) <0.001	1.65 (1.37.1.98) <0.001	1.58 (1.31.1.91) <0.001	1.46 (1.2.1.79) <0.001
HMGB1 (categorical)					
Negative	24 (38.7)	1	1	1	1
Low	33 (52.4)	1.74 (0.86.3.55)0.126	1.67 (0.81.3.41)0.163	1.49 (0.72.3.12)0.285	1.39 (0.66.2.95)0.387
Moderate	38 (60.3)	2.41 (1.17.4.94)0.017	2.33 (1.13.4.8)0.021	1.95 (0.93.4.12)0.079	1.5 (0.69.3.28)0.306
High	58 (92.1)	18.37 (6.45.52.32) <0.001	15.71 (5.35.46.12) <0.001	11.79 (3.94.35.29) <0.001	7.84 (2.52.24.36) <0.001
*p*-value for trend		<0.001	<0.001	<0.001	0.005

Model I Unadjusted; Model II Adjusted for age; Model III Adjusted for age, HE, infection, and ascites; Model IV Adjusted for age, HE, infection, ascites, Na, TBIL, and INR.

As shown in Supplementary Figure S4 **,** to further assess the role of HMGB1 levels in AKI development in HBV-ACLF individuals, the subgroup results were analyzed. Interactions were observed in all subgroups, including age, cirrhosis, HE, infection, ascites, TBIL, INR, sCr, CysC, lactate, MELD, Child-Pugh and CLIF-C ACLF scores except Na.

### Potential prognostic value of HMGB1 for AKI in individuals with ACLF

**FIGURE 5:**
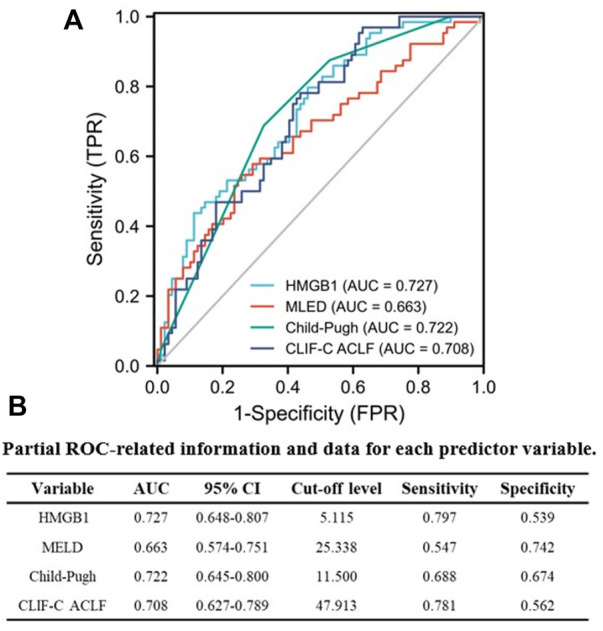
Performance of serum HMGB1 level, MELD, Child-Pugh and CLIF-C ACLF scores were compared in predicting 90-day adverse outcomes in AKI patients using AUROC **(A)**. **(B)** Partial ROC-related information and data for each predictor variable (AUROC, area under the receiver operating characteristic curve; AKI, acute kidney injury; HMGB1, high-mobility group box 1; MELD, model for end-stage liver disease; Child-Pugh, Child-Turcotte-Pugh; CLIF-C ACLF; CLIF-Consortium ACLF).

Next, we explored the prognostic value of HMGB1 in AKI patients, and an AUROC analysis was used to compare the performance of HMGB1, MELD, Child-Pugh and CLIF-C ACLF scores separately according to 90-day prognosis. From Figure 5A, discrimination was relatively higher for HMGB1 [0.727 (95% CI: 0.648–0.807) than the AUROC of the MELD [0.663 (95% CI: 0.574–0.751)], Child-Pugh [0.722 (95% CI: 0.645–0.800)], and CLIF-C ACLF [0.708 (95% CI: 0.627–0.789)] scores, although these differences did not reach statistical significance. Partial ROC-related information and data for each predictor variable are listed in Figure 5B. Furthermore, we further explored the potential prognostic value of HMGB1 in the non-cirrhotic (n = 68) and cirrhotic subgroups (n = 85) of all AKI patients. It is interesting that the AUROC of HMGB1 was above 0.7 in the cirrhotic subgroup [0.800 (95% CI: 0.706–0.894)] and below 0.7 in the non-cirrhotic subgroup [0.644 (95% CI: 0.512–0.776)] (Supplementary Figure S5 and Supplementary Table S5).

Furthermore, multivariate regression analyses were performed to identify independent risk factors related to 90-day poor outcomes of all AKI individuals. In unadjusted or adjusted models, we found an association between HMGB1 level as a continuous or categorical variable and 90-day prognosis. Importantly, the risk of 90-day adverse outcomes increased with higher HMGB1 values (*p*-value for trend = 0.004) in Model Ⅳ adjusted for age, infection, and CLIF-C ACLF (Supplementary Table S6).

To further assess the role of HMGB1 levels on 90-day outcomes of AKI individuals, the subgroup results were analyzed. According to the 90-day prognosis, interactions were observed in all subgroups, including age, cirrhosis, HE, infection, ascites, AKI stage, TBIL, INR, sCr, lactate, MELD, Child-Pugh and CLIF-C ACLF scores (*p* < 0.05 Supplementary Figure S6).

## Discussion

AKI is a common and life-threatening complication in end-stage liver disease. The 30-day mortality in AKI patients with ACLF remains very high (approximately 50%) (Maiwall et al., 2017). The early diagnosis and timely intervention of AKI in ACLF is extremely important to improve prognosis. Circulating HMGB1 has pleiotropic physiologic effects in several diseases and has potential as a biomarker for relevant diseases. Our study investigated the role of HMGB1 in AKI in individuals with HBV-ACLF. There were three major findings in our study. First, AKI is quite common among patients with HBV-ACLF. Patients with AKI have a significantly worse prognosis than those without AKI. Elevated HMGB1 levels are associated with AKI stage in patients with HBV-ACLF. Second, HMGB1 can be used as an early AKI predictor in patients with ACLF. Finally, serum HMGB1 can serve as a potential prognostic biomarker for AKI in individuals with HBV-ACLF. HMGB1 is a highly conserved nucleoprotein implicated in several bioactivities. During the past decade, many researchers have proposed that HMGB1 acts as a key signaling molecule in kidney and liver diseases (Chen et al., 2014; Chen Q et al., 2016; Zhao et al., 2020). Among patients with HBV-ACLF, high levels of HMGB1 correlate closely with coagulation and brain injury. A recent study also found that HMGB1 levels were significantly increased in patients with HE after transjugular intrahepatic portosystemic shunt (Chen et al., 2020). However, we found no association between HMGB1 levels and liver injury, which may be due to inherent differences in the populations studied. However, Cai J et al. found higher HMGB1 expression in HBV-ACLF patients (Cai et al., 2016).

Previous studies have suggested that CysC can be used for early prediction of AKI (Hou et al., 2021; Ye et al., 2021; Galic et al., 2022), including in patients with HBV-ACLF complicated by AKI (Wan et al., 2013). A recent study demonstrated that HMGB1 is a potential preprocedural predictor of contrast-induced AKI after percutaneous coronary artery intervention (Mo et al., 2022). When compared to CysC, serum HMGB1 had a stronger predictive potential for AKI or severe AKI (relatively higher AUC values). The most meaningful AUC value for the joint indicator (HMGB1+CysC) had excellent sensitivity (0.724) and specificity (0.706) for predicting AKI and sensitivity (0.947) and specificity (0.500) for predicting severe AKI (Supplementary Table S3). On multivariable analysis, baseline HMGB1 was an independent predictor of AKI in HBV-ACLF patients after adjusting for all confounding factors. Multiple studies have shown that serum HMGB1 is predictive of the outcomes of end-stage liver and renal disease (Leelahavanichkul et al., 2011; Zhang et al., 2014; Dai et al., 2021; Dos Santos Pinheiro et al., 2022). Here, we showed that serum HMGB1 might be a prognostic biomarker for AKI among HBV-ACLF patients, while it was superior in the cirrhotic subgroup. HMGB1, infection, and Child-Pugh score are considered to be independent risk factors for 90-day adverse clinical outcomes of AKI among HBV-ACLF patients. A large retrospective multicenter study of 1032 patients showed that the MELD score, ascites, sepsis/infection, and acute variceal bleeding were independent risk factors predicting the development of AKI in ACLF patients with underlying cirrhosis in China (Zang et al., 2016). Concurrently, elevated serum HMGB1 levels appear to be clearly associated with sterile inflammation and infection (Kang et al., 2017). In summary, we believe that the serum HMGB1 level shows great value in facilitating clinical decision-making for the clinical management of patients with HBV-ACLF complicated with AKI. In our study, patients with AKI had a greater risk of complications such as HE, infection, and ascites than those without AKI. These findings were similar to our previous study (Yuan et al., 2017), which suggested that spontaneous bacterial peritonitis had some prognostic value for patients with AKI (Yuan et al., 2017). Therefore, early proactive intervention for complications can significantly improve the prognosis of ACLF-associated AKI patients.

In addition, ACLF-associated AKI may involve not only tissue hypoperfusion but, more importantly, capillary leukocyte infiltration, vascular microthrombosis, and cell apoptosis (Zaccherini et al., 2021). HMGB1 is a non-histone chromosomal protein known to regulate gene transcription and DNA repair by binding to DNA or chromatin *via* receptor for advanced glycation end-products (RAGE) and Toll-like receptor 4 (TLR4). One clinical study showed that HMGB1, RAGE and interleukin (IL)-17 expression is increased in the liver tissue of HBV-ACLF patients, and the HMGB1 and RAGE interaction may contribute to inflammation of the liver, enhancing the expression of IL-17 (Jhun et al., 2015). Intriguingly, a recent experimental study showed that compared with wild-type control mice, mice lacking both RAGE and soluble isoforms (sRAGE) had more severe AKI, with enhanced renal tubular damage, macrophage infiltration, and fibrosis (Miyagawa et al., 2022). However, additional mechanisms or pathways are certainly involved, for example, autophagy-mediated HMGB1 release or the novel epigenetic role of HMGB1. The precise molecular mechanisms remain to be elucidated in future work. Increasing evidence indicates that HMGB1 targeting is a potential AKI therapy. A more recent animal study found that ulinastatin combined with thrombomodulin significantly improved LPS-induced liver and kidney pathological structure and functional injury; additionally, these improvements in survival of endotoxic rats by inhibiting liver and kidney cell apoptosis, promoting proliferation, and inhibiting inflammation and oxidative injury (Zhang et al., 2022). To date, numerous scholars have concentrated on HMGB1 neutralizing antibodies, and cytokines released by immune cells can be reduced by inhibiting HMGB1-TLR4 binding *in vitro* (Venereau et al., 2012). In drug-induced acute liver failure and ACLF models, there were significant decreases in serum HMGB1 levels and a reduction in liver injury after intervention with the anti-HMGB1 antibody, which ultimately contributed to the significantly improved animal survival (Takano et al., 2010; Li et al., 2013). Nevertheless, translation of these promising findings into the clinical realm remains to be done.

Nevertheless, despite many strengths, the current study has some possible limitations. First, the data of our cohort were collected in a consistent fashion. However, this is a single-center study; therefore, the results should be validated in multicenter studies. In addition, we analyzed HMGB1 levels only at baseline, and we were unable to evaluate dynamic changes in HMGB1 levels over time. Finally, the design of our study does not allow us to draw mechanistic conclusions about the role of HMGB1 in the underlying pathophysiology of ACLF. Further studies are required to elucidate the pathophysiological and molecular regulatory mechanism of HMGB1 in ACLF-associated AKI.

## Conclusion

In conclusion, our study suggests that elevated HMGB1 levels are implicated in AKI stage in HBV-ACLF patients and that the determination of serum HMGB1 levels may be of important early prediction and prognostic implications in HBV-ACLF patients with AKI. To confirm our findings, further investigation in larger real-world studies is warranted to confirm the clinical significance and prognostic value of HMGB1 in ACLF-associated AKI.

## Data Availability

The raw data supporting the conclusions of this article will be made available by the authors, without undue reservation.
