# Supplementary figures and images for: Determination of HMGB1 in hepatitis B virus-related acute-on-chronic liver failure patients with acute kidney injury: Early prediction and prognostic implications
Source: Front Pharmacol. 2023 Jan 13;13:1031790. doi: 10.3389/fphar.2022.1031790 (PMC9880762; doi:10.3389/fphar.2022.1031790)

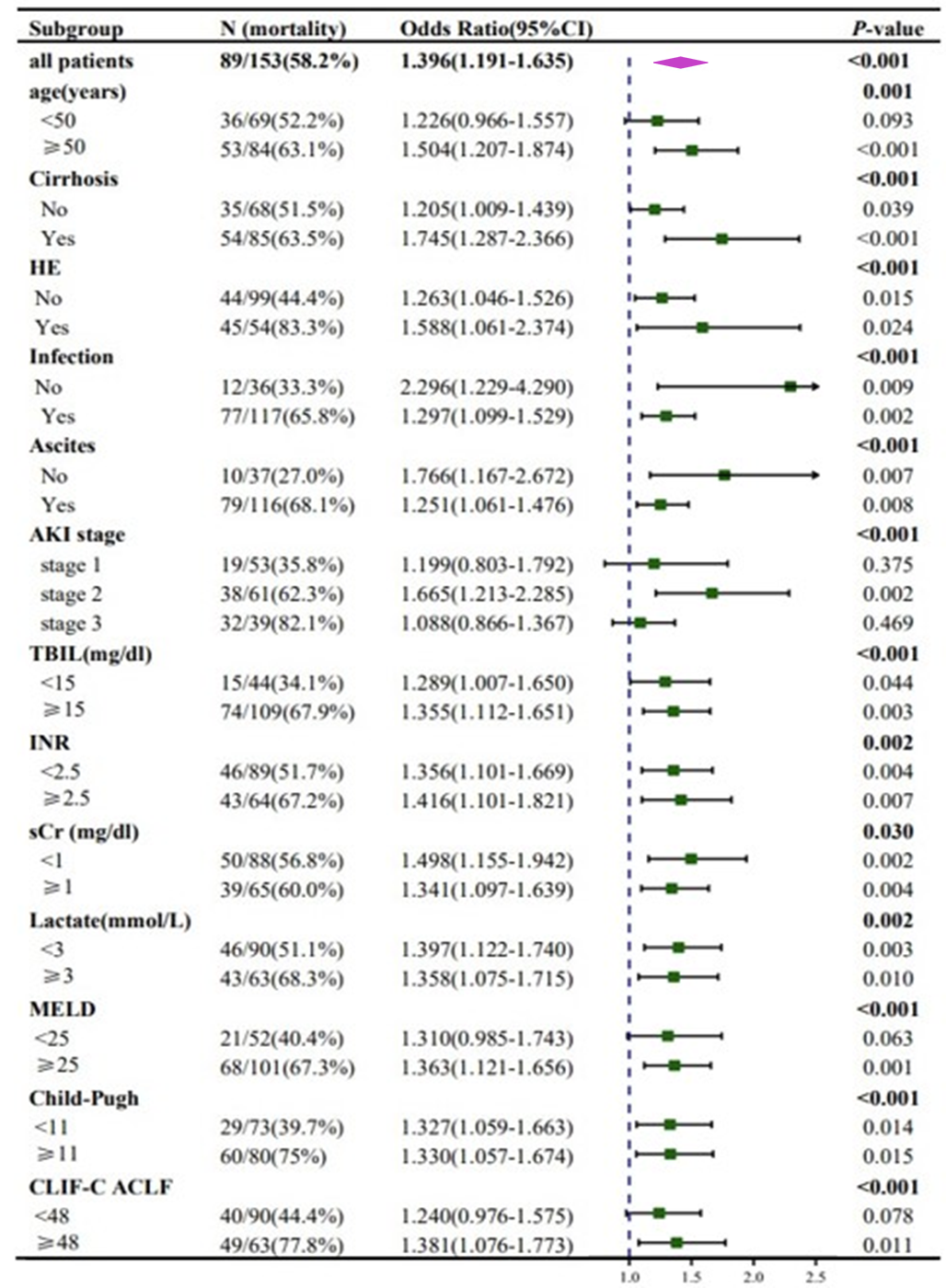

Supplement: Supplementary file 1 [file Image6.tif]

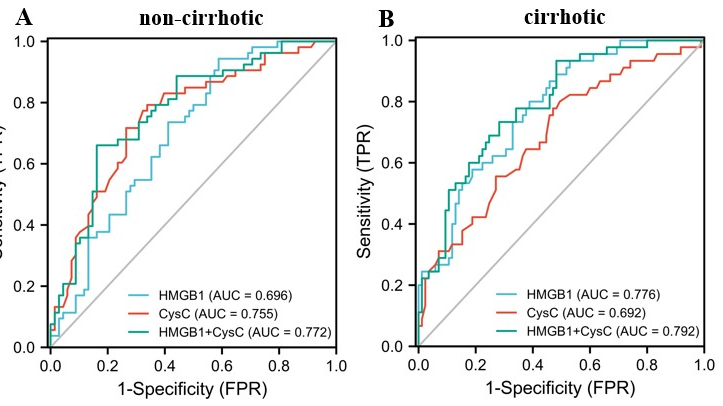

Supplement: Supplementary file 3 [file Image3.tif]

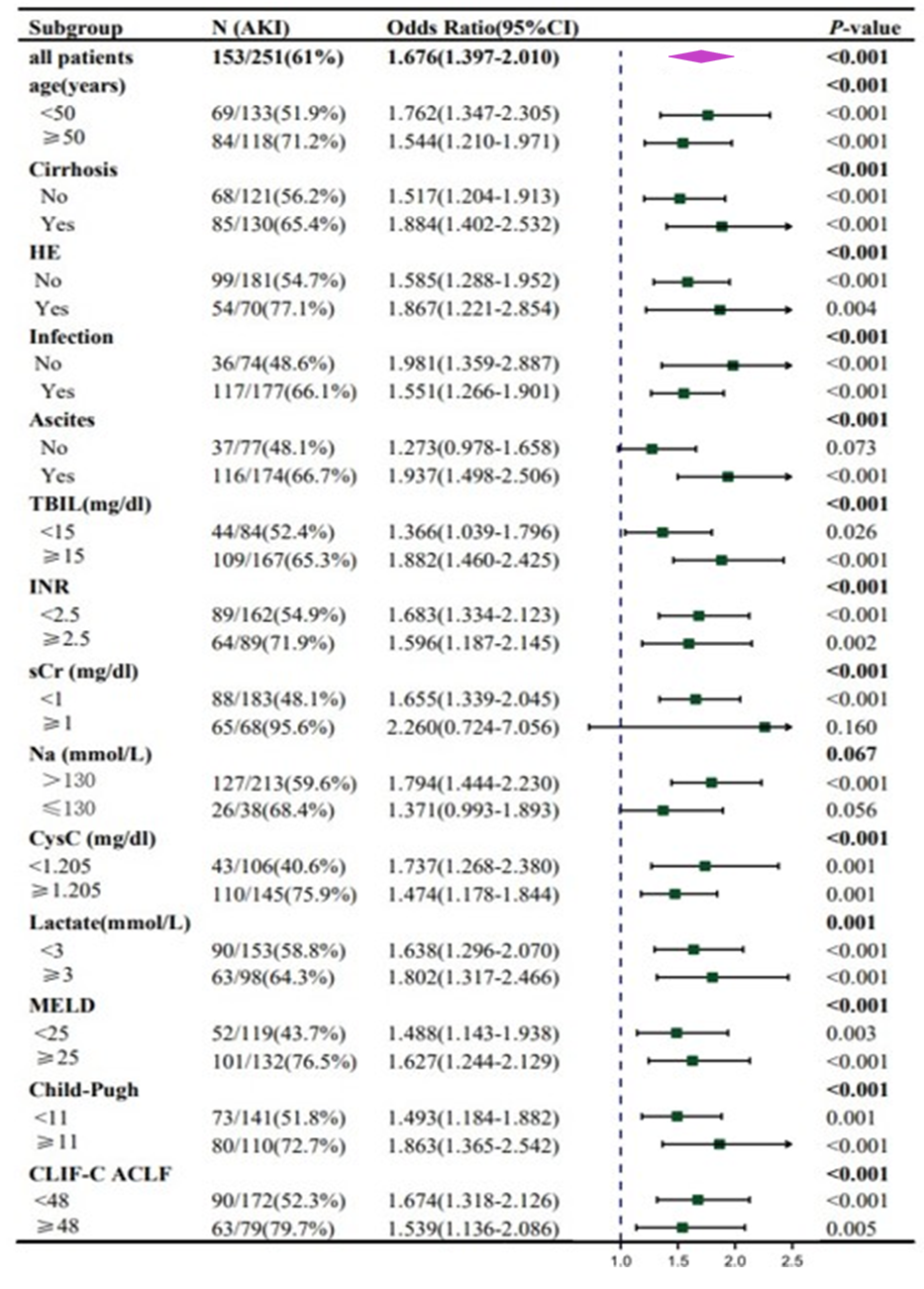

Supplement: Supplementary file 4 [file Image4.tif]

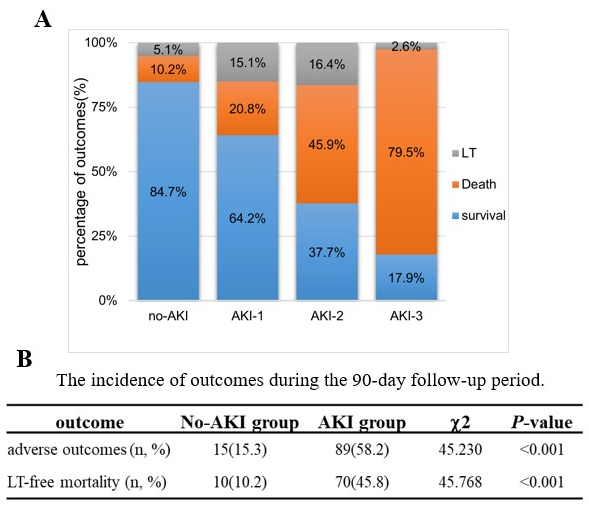

Supplement: Supplementary file 5 [file Image2.tif]

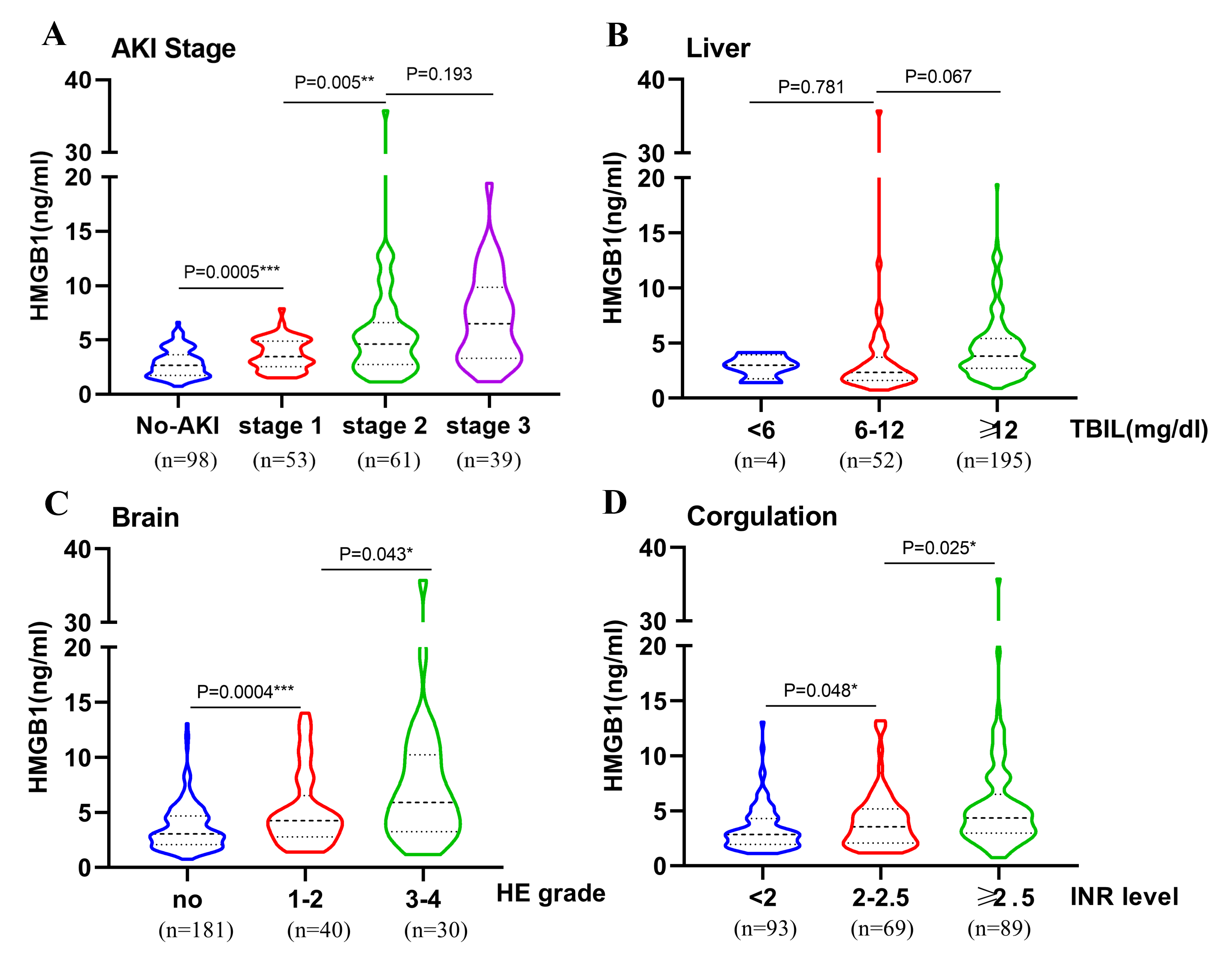

Supplement: Supplementary file 6 [file Image1.tif]

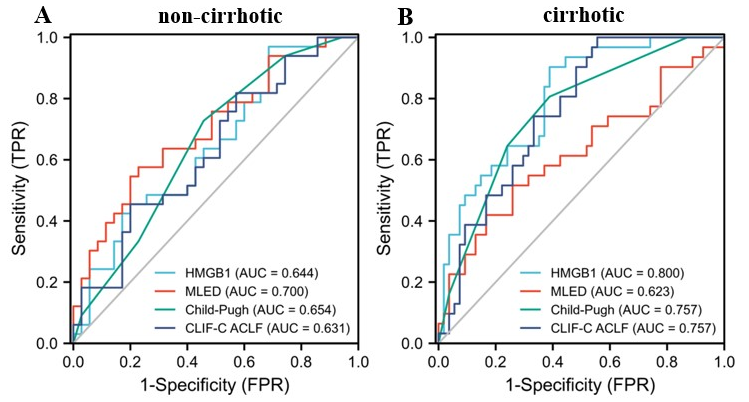

Supplement: Supplementary file 7 [file Image5.tif]
