# Supplementary material for: Determination of HMGB1 in hepatitis B virus-related acute-on-chronic liver failure patients with acute kidney injury: Early prediction and prognostic implications
Source: Front Pharmacol. 2023 Jan 13;13:1031790. doi: 10.3389/fphar.2022.1031790 (PMC9880762; doi:10.3389/fphar.2022.1031790)
Supplement: Supplementary file 2 [file Table1.pdf]

Supplementary Material

1 Supplementary Methods

1.1 Scoring

The MELD score was calculated as MELD = 9.57 × ln[creatinine (mg/dl)] + 3.78 × ln[TBIL (mg/dl)] + 11.2 × ln(INR) + 6.4 [1].

The Child–Pugh score was calculated from the serum TBIL level, albumin level, HE, ascites volume, and prothrombin time [2].

The CLIF-C OF score was used for the identification of organ failure. It was calculated from Bilirubin, Creatinine, Encephalopathy, INR, MAP, Oxygenation (PaO_2/FiO_2)[3].

The CLIF-C ACLF score was calculated based on the CLIF Consortium Organ Function (CLIF-OF) score as follows: CLIF-C-ACLF = 10 × [0.33 × CLIF-OF + 0.04 × age + 0.63 × ln (white blood cell count [WBC]) – 2] [3].

1. Kamath PS, Kim WR. The model for end-stage liver disease (MELD). Hepatology (Baltimore, Md). 2007;45(3):797-805.
2. Cholongitas E, Papatheodoridis GV, Vangeli M, Terreni N, Patch D, Burroughs AK. Systematic review: The model for end-stage liver disease--should it replace Child-Pugh's classification for assessing prognosis in cirrhosis? Aliment Pharmacol Ther. 2005;22(11-12):1079-1089.
3. Jalan R, Saliba F, Pavesi M, et al. Development and validation of a prognostic score to predict mortality in patients with acute-on-chronic liver failure. J Hepatol. 2014;61(5):1038-1047.

2 Supplementary Figure and Table Legends

2.1 Supplementary Figure Legends

Supplementary Figure 1 Serum HMGB1 levels of different stages of AKI and comparisons of serum HMGB1 levels among patients with various degrees of organ injury according to CLIF-OF score. (A) AKI stage, (B) liver, (C) brain, and (D) coagulation.

(HMGB1, high-mobility group box 1; CLIF-OF, CLIF Consortium Organ Failure Score; **P* < 0.05; ***P* < 0.01; ****P* < 0.001)

Supplementary Figure 2 A significant survival advantage was observed at 90 days in the no-AKI group. A: The percentage of 90-day different outcomes in the no-AKI and AKI stage 1/2/3 groups. B: The incidence of outcomes during the 90-day follow-up period.

Supplementary Figure 3 Performance of serum CysC and HMGB1 levels were compared in predicting AKI development within the non-cirrhotic (n = 121) or cirrhotic subgroup (n = 130) of HBV-ACLF patients using AUROC.

(AUROC, area under the receiver operating characteristic curve; AKI, acute kidney injury; HMGB1, high-mobility group box 1)

Supplementary Figure 4 Effects of HMGB1 on AKI development in subgroups of patients with AKI.

Supplementary Figure 5 Performance of MELD, Child-Pugh, CLIF-C ACLF scores and serum HMGB1 levels were compared in predicting 90-day adverse outcomes within the non-cirrhotic (n = 68) or cirrhotic subgroup (n = 85) of AKI patients using AUROC.

(AUROC, area under the receiver operating characteristic curve; AKI, acute kidney injury; HMGB1, high-mobility group box 1; MELD, model for end-stage liver disease; Child-Pugh, Child-Turcotte-Pugh; CLIF-C ACLF, CLIF-

Consortium ACLF)

Supplementary Figure 6 Effects of HMGB1 on 90-day adverse outcomes in subgroups of patients with AKI.

2.2 Supplementary Table Legends

Supplementary Table 1 Kidney outcomes of different maximum AKI stages in the total AKI population during the 90-day follow-up period.

Maximum AKI	Frequency (n, %)	CR (n, %)	AKI Stage 1 (n, %)	AKI Stage 2 (n, %)	AKI Stage 3 (n, %)
Stage 1 (0.3)	17(11.1)	13(76.5)	4(23.5)	NA	NA
Stage 1 (50%)	36(23.5)	21(58.3)	15(41.7)	NA	NA
Stage 2	61(39.9)	19(31.1)	10(16.4)	32(52.5)	NA
Stage 3	30(19.6)	2(6.7)	0(0)	2(6.7)	26(86.7)
Stage 3(RRT)	9(5.9)	2(22.2)	2(22.2)	2(22.2)	3(33.3)
Total	153(100.0)	57(37.3)	31(20.3)	36(23.5)	29(19)

CR, complete response.

Supplementary Table 2 Kidney outcomes of different AKI stages in survivors by using ICA criteria during the 90-day follow-up period.

Maximum AKI	Frequency (n, %)	ICA (n, %)		
		CR	PR	AR
Stage 1 (0.3)	11(17.2)	8(72.7)	NA	3(27.3)
Stage 1 (50%)	23(35.9)	17(73.9)	NA	6(26.1)
Stage 2	23(35.9)	13(56.5)	5(21.7)	5(21.7)
Stage 3	3(4.7)	1(33.3)	1(33.3)	1(33.3)
Stage 3(RRT)	4(6.3)	2(50)	2(50)	0(0)
Total	64(100.0)	41(64.1)	8(12.5)	15(23.4)

AR, absence of response; CR, complete response; PR, partial response; ICA, International Club of Ascites; NA, not applicable.

Supplementary Table 3 Partial ROC-related information and data for each predictor variable for the development of AKI(Figure 3A) or severe AKI(Figure 3B).

Variable	AUC	95% CI	Cut-off level	Sensitivity	Specificity
**AKI(n=153)**					
HMGB1	0.740	0.680-0.800	4.850	0.939	0.444
CysC	0.728	0.664-0.791	1.205	0.643	0.719

HMGB1+CysC	0.772	0.715-0.830	-0.238	0.724	0.706
**severe AKI(n=100)**					
HMGB1	0.746	0.680-0.812	5.301	0.934	0.490
CysC	0.744	0.682-0.806	1.335	0.709	0.690
HMGB1+CysC	0.785	0.724-0.845	-0.165	0.947	0.500

Supplementary Table 4 Partial ROC-related information and data for each predictor variable for AKI development within the non-cirrhotic (n = 121) and cirrhotic (n = 130) subgroups of all HBV-ACLF individuals (n=251).

Variable	AUC	95% CI	Cut-off level	Sensitivity	Specificity
**non-cirrhotic subgroup (n=121)**					
HMGB1	0.696	0.604-0.789	4.789	0.943	0.412
CysC	0.755	0.667-0.842	1.305	0.792	0.662
HMGB1+CysC	0.772	0.687-0.856	0.178	0.660	0.838
**cirrhotic subgroup (n=130)**					
HMGB1	0.776	0.696-0.856	3.766	0.800	0.612
CysC	0.692	0.596-0.788	1.410	0.778	0.529
HMGB1+CysC	0.792	0.714-0.870	-1.210	0.933	0.518

Supplementary Table 5 Partial ROC-related information and data for each predictor variable for 90-day adverse outcomes within the non-cirrhotic (n = 68) and cirrhotic (n = 85) subgroups of all AKI individuals.

Variable	AUC	95% CI	Cut-off level	Sensitivity	Specificity
**non-cirrhotic subgroup (n=68)**					
HMGB1	0.644	0.512-0.776	7.611	0.970	0.314
MELD	0.700	0.576-0.825	25.338	0.576	0.771
Child-Pugh	0.655	0.527-0.781	11.500	0.727	0.543
CLIF-C ACLF	0.631	0.498-0.764	41.356	0.455	0.800
**cirrhotic subgroup (n=85)**					
HMGB1	0.800	0.706-0.894	5.115	0.903	0.611
MELD	0.623	0.493-0.754	25.239	0.516	0.741
Child-Pugh	0.757	0.658-0.857	12.500	0.806	0.611
CLIF-C ACLF	0.757	0.657-0.858	54.347	1.000	0.444

HMGB1, high-mobility group box 1; Child-Pugh, Child-Turcotte-Pugh; MELD, model for end-stage liver disease; CLIF-C ACLF, CLIF- Consortium ACLF.

Supplementary Table 6 Adjusted effects of HMGB1 level on 90-day adverse outcomes in AKI patients (n=153).

HMGB1	Number of 90-day adverse outcomes (n, %)	OR, 95% CI, *P*- value<br>Model I	OR, 95% CI, *P*- value<br>Model II	OR, 95% CI, *P*- value<br>Model III	OR, 95% CI, *P*- value<br>Model IV
**HMGB1 (continuous)**	89(58.17)	1.4(1.19,1.64) <0.001	1.38(1.17,1.63) <0.001	1.37(1.15,1.62) <0.001	1.28(1.07,1.53)0.007
**HMGB1 (categorical)**					
**Negative**	10(26.3)	1	1	1	1
**Low**	26(66.7)	5.6(2.1,14.95)0.001	5.61(2.1,15.01)0.001	5.63(2.04,15.52)0.001	5.44(1.92,15.43)0.001
**Moderate**	21(55.3)	3.46(1.32,9.07)0.012	3.42(1.3,8.99)0.013	4(1.45,11.04)0.007	3.14(1.1,9)0.033
**High**	32(84.2)	14.93(4.81,46.33) <0.001	12.97(3.99,42.16) <0.001	10.81(3.24,36.03) <0.001	6.79(1.94,23.81)0.003
***P* value for trend**		<0.001	<0.001	<0.001	0.004

Model I Unadjusted;

Model II Adjusted for age;

Model III Adjusted for age, and infection;

Model IV Adjusted for age, infection, and CLIF-C ACLF.
